# A Colorimetric/Fluorescent Dual-Mode Aptasensor for *Salmonella* Based on the Magnetic Separation of Aptamers and a DNA-Nanotriangle Programmed Multivalent Aptamer

**DOI:** 10.3390/foods12203853

**Published:** 2023-10-20

**Authors:** Na Ma, Mengni Sun, Hanxing Shi, Liangliang Xue, Min Zhang, Wenge Yang, Yali Dang, Zhaohui Qiao

**Affiliations:** 1 College of Food and Pharmaceutical Sciences, Ningbo University, Ningbo 315800, China; 2 Zhejiang-Malaysia Joint Research Laboratory for Agricultural Product Processing and Nutrition, Ningbo University, Ningbo 315800, China

**Keywords:** *Salmonella* detection, multivalent aptamer, colorimetric/fluorescent dual mode, DNA nanotriangle

## Abstract

*Salmonella* infection has emerged as a global health threat, causing death, disability, and socioeconomic disruption worldwide. The rapid and sensitive detection of *Salmonella* is of great significance in guaranteeing food safety. Herein, we developed a colorimetric/fluorescent dual-mode method based on a DNA-nanotriangle programmed multivalent aptamer for the sensitive detection of *Salmonella*. In this system, aptamers are precisely controlled and assembled on a DNA nanotriangle structure to fabricate a multivalent aptamer (NTri-Multi-Apt) with enhanced binding affinity and specificity toward *Salmonella*. The NTri-Multi-Apt was designed to carry many streptavidin-HRPs for colorimetric read-outs and a large load of Sybr green I in the dsDNA scaffold for the output of a fluorescent signal. Therefore, combined with the magnetic separation of aptamers and the prefabricated NTri-Multi-Apt, the dual-mode approach achieved simple and sensitive detection, with LODs of 316 and 60 CFU/mL for colorimetric and fluorescent detection, respectively. Notably, the fluorescent mode provided a self-calibrated and fivefold-improved sensitivity over colorimetric detection. Systematic results also revealed that the proposed dual-mode method exhibited high specificity and applicability for milk, egg white, and chicken meat samples, serving as a promising tool for real bacterial sample testing. As a result, the innovative dual-mode detection method showed new insights for the detection of other pathogens.

## 1. Introduction

Foodborne diseases have emerged as a growing global concern [1]. *Salmonella*, as the most widespread foodborne bacterial pathogen, has caused extensive food poisoning, illness, and death. It is reported that worldwide, more than 100 million people are infected and 370,000 people die from *Salmonella* each year [2]. The World Health Organization (WHO) estimates that there are more than 16 million global infections caused by *Salmonella* per year [3]. Even worse, if the infections are not treated in time, the final mortality rate can reach 10% [4]. The sensitive and reliable detection of *Salmonella* is pivotal for effectively mitigating its contamination and curtailing its further propagation within the food supply chain [5,6] Conventional *Salmonella* detection strategies are mainly based on microbiological cultures, polymerase chain reaction (PCR)-based methods, and immunological methods. As the gold-standard method, microbiological cultures are time-consuming (lasting 4–7 days) and laborious, which is unsuitable for rapid screening and field applications. The PCR-based nucleic acid analysis exhibits remarkable sensitivity and specificity; however, it involves intricate procedural steps, demands technical expertise, and necessitates specialized equipment [7]. On the other hand, immunological methods have the advantages of high levels of specificity, low costs, and automatic operation, but the sensitivity is unsatisfactory (10^4^ CFU/mL) [8]. Therefore, there remains a significant demand for the development of a sensitive, simple, and rapid method of detection for *Salmonella*.

Aptamers, called chemical antibodies, have emerged as highly promising recognition element candidates in the field of bacterial detection [9]. These aptamers are single-stranded nucleic acid oligomers that exhibit the ability to bind specifically to their target molecules through the formation of unique secondary or tertiary structures [10]. In comparison to antibodies, aptamers offer numerous distinctive advantages, including exceptional chemical stability, cost-effectiveness, facile synthesis and modification, and batch-to-batch consistency [11]. These superior advantages enable the extensive application of aptamers in the detection of *Salmonella* [12,13]. However, aptamers remain challenged in the bacterial detection of real samples due to the diminished affinity induced by food matrixes [14]. Fortunately, the multivalent effect has been investigated to improve the reduced affinity of aptamers [15]. Multivalent aptamers assemble aptamer monomers onto scaffolds, such as nanoparticles [16], liposomes [17], or DNA nanostructures [18], thereby increasing the local aptamer density and facilitating synergistic binding, ultimately resulting in significant enhancements of avidity and specificity. Among these scaffolds, DNA nanostructures, such as DNA cubes [19], pentacles [20], and triangular pyramid frustums [21], have been reported with much higher affinities than monovalent aptamers and applied for the detection of pathogens. Thus, a DNA-nanostructure-based multivalent aptamer shows great potential in the sensitive detection of bacteria.

Colorimetric detection is one of the most attractive optical assays due to its merits of simple operation, cost-effective analysis, rapid results, and the lack of a need for a complicated apparatus [22]. Nevertheless, the sensitivity is poor and susceptible to interferences from changes in the operating conditions and biological environments. In contrast, fluorescent detection assays exhibit heightened sensitivity and superior resilience against external interferences [23]. Consequently, an innovative approach involving the integration of colorimetric and fluorescence modalities was conceived which aims to augment sensitivity, stability, and detection precision by leveraging the distinct virtues of each sensing mode [24]. This dual-mode detection finds applications in many fields, including medical diagnostics [25], environmental surveillance [26], and food analyses [27]. Unfortunately, the majority of dual-mode detection approaches rely upon intricate functional nanomaterials which always involve complicated preparation and extreme conditions (such as toxic reagents and high temperatures and pressures) [28], consequently leading to their instability [29]. In light of these challenges, there emerges an imperative to introduce facile labeling moieties capable of streamlining synthetic procedures while concurrently conferring robustness upon the dual-mode detection scheme.

Herein, we developed a dual-readout method for the detection of *Salmonella* based on a colorimetric-tag- and fluorescent-tag-labeled DNA-nanotriangle programmed multivalent aptamer. In this study, the aptamer was precisely controlled and assembled on a DNA nanotriangle structure to fabricate a multivalent aptamer (NTri-Multi-Apt) with enhanced binding affinity and specificity toward *Salmonella*. In addition to labeling the target bacteria with high affinity, the NTri-Multi-Apt can amplify the signal via the tag carried on its scaffold and output a visual colorimetric read-out. Furthermore, the double-stranded DNA nanotriangle loaded a large amount of Sybr green I for the generation of a fluorescence signal which provides self-calibration and an improved sensitivity compared to colorimetric detection. Combined with the magnetic separation of the aptamer, the simple, sensitive, and specific detection of *Salmonella* in food samples can be achieved based on the DNA-nanotriangle multivalent-aptamer-assisted colorimetric/fluorescent dual-readout method.

## 2. Materials and Methods

### 2.1. Materials and Apparatuses

Streptavidin-coated magnetic nanoparticles (SA-MNPs) were obtained from Ocean NanoTech (San Diego, CA, USA). Sybr green I was obtained from Meilun Bio technology Co., Ltd. (Dalian, China); BSA, 4 S GelRed, agarose, TAE buffer, and SA-HRP were sourced from Sangon Biotech Co., Ltd. (Shanghai, China); and 2 K Plus DNA Marker was obtained from Tsingke Biotechnology Co., Ltd. (Beijing, Chian). Luria broth (LB) was provided by Hangzhou Microbial Reagent Co., Ltd. (Hangzhou, China). TMB was supplied by Real-Times Biotechnology Co., Ltd., (Beijing, China) and brain heart infusion broth (BHI) was acquired from Qingdao Hope Bio-Technology Co., Ltd. (Qingdao, China). A 1 × TMS buffer was composed of 10 mM of Tris and 80 mM of MgCl_2_. The pH of the 1 × TMS buffer was adjusted to 7.5 using glacial acetic acid. All the buffers above were prepared using ultrapure water (>18.25 MΩ cm) from a Milli-Q^®®^ water purification system (Millipore, Billerica, MA, USA). All oligonucleotide sequences (Tables S1–S4) were synthesized by Sangon Biotech (Shanghai, China). The optical absorbance and fluorescence intensities were determined using a SpectraMax i3 Multi-Mode Microplate Reader. The fluorescence was viewed under a Blue Light LED Transilluminator (Shanghai Jiapeng Technology Co., Ltd., Shanghai, China). Electrophoresis images were captured and analyzed using a Bio-Rad Gel Doc XR+ system (Bio-Rad, Richmond, CA, USA). The fluorescent microscope photographs were obtained via a confocal laser scanning microscope (CLSM) (Leica, Solms, Germany).

### 2.2. Bacterial Culture

The typical representative of *Salmonella* we chose was *Salmonella* typhimurium (ATCC 14028). *Salmonella* enteritidis (CICC 10982), *E. coli O157:H7* (ATCC 43889), *Listeria monocytogenes* (CICC 21540), *Vibrio parahaemolyticus* (CICC 21617)*, S. aureus* (ATCC 25923), *Vibrio parahaemolyticus* (ATCC 17802), *Vibrio alginolyticus* (ATCC 17749)*,* and *Vibrio vulnificus* (ATCC 27562) were used for specific detections and cultured in an LB liquid culture medium for 20 h at 37 °C.

Before its utilization, the bacterial solution underwent centrifugation, followed by resuspension in PBS subsequent to a PBST wash. Subsequently, the bacterial solution was subjected to a 10-fold dilution in PBS to yield samples with varying concentrations spanning from 10^2^ to 10^7^ colony-forming units per milliliter (CFU/mL). To determine the bacterial counts per milliliter, the *Salmonella* strain was plated on agar plates. The colonies were counted to obtain the CFUs per milliliter after incubation. To ensure sterilization, all receptacles used for bacterial handling were subjected to disinfection in an autoclave before usage.

### 2.3. The Preparation of Aptamer-Functionalized Magnetic Nanoparticles (MNP-Apts)

MNP-Apts were synthesized based on the classical reaction between streptavidin-modified MNPs (SA-MNPs) and biotin-modified aptamers (biotin-Apts). Briefly, 100 μL of 1 mg/mL SA-MNPs and 100 μL of 1 μM biotin-Apts were mixed and incubated at RT for 30 min. The complexes were then supplied with an equal volume of D-biotin (4 μM) at RT for 30 min to block the unbound sites of the SA on the surfaces of the MNPs. Subsequently, the product was separated with the assistance of a magnet and washed with PBST buffer three times. Finally, the obtained MNP-Apts were resuspended in 100 μL of PBS buffer and stored at 4 °C until further use.

### 2.4. The Preparation and Characterization of the NTri-Multi-Apt

Before the reaction, all DNA sequences were solubilized in 1 × TMS, leading to a final molarity of 50 μM. The NTri-Multi-Apt was prepared by mixing equimolar amounts of four scaffold clip strands (a, b, c, L) in 1 × TMS buffer. The solution was heated to 95 °C for 4 min and rapidly cooled to 4 °C to fabricate the NTri-Multi-Apt. Subsequently, the removal of free strands was carried out through ultrafiltration to obtain a pure NTri-Multi-Apt. Then, SA-HRP (0.5 nM) was added to combine the biotin group of aptamers, and 2 × Sybr green I was incubated to embed it in the dsDNA at RT for 25 min. As a result, a dual-mode NTri-Multi-Apt was synthesized and stored at 4 °C for further use. NTri-monovalent and divalent aptamers were prepared using the same method with the selective substitution of specific ssDNA sequences (Figure S1, Table S1).

The NTri-Multi-Apts were characterized via agarose gel electrophoresis in 1 × TAE buffer at 130 V for 30 min. The loading sample was prepared by mixing 5 μL of DNA samples with 1 μL of loading buffer. In addition, the shape of the NTri-Multi-Apt was observed via atomic force microscopy (AFM), using 1 μM of NTri-Multi-Apts as an analytical sample.

### 2.5. The Binding Affinity of the NTri-Multi-Apt

In this study, the binding affinities of the NTri-Multi-Apts were analyzed via an ELISA. Briefly, the plates were coated with a *Salmonella* solution at 37 °C for 2 h and blocked with 1% BSA. After washing them with PBST, different concentrations of the NTri-triApt (0.01–0.1 μM) were added to capture the target. After washing out unbound aptamers three times using PBST, SA-HRP was added to combine the aptamers on *Salmonella*. Then, the color reaction was initiated by adding 50 μL of TMB. Then, the absorbance intensity (450 nm) was measured using a Microplate Reader. Finally, the Kd values were determined via Y = Bmax X/(Kd + X) (where X represents the molarity of the NTri-triApt, Y indicates the optical density, and Bmax represents the maximum Y achieved during the detection process). Similarly, the affinities of NTri-monoApt and NTri-biApt were also calculated using the same method described above. To gain further insight into the enhanced affinity of the NTri-triApt, we employed confocal microscopy to observe *Salmonella* labeled with NTri-triApt, NTri-biApt, and NTri-monoApt, all of which were modified via FAM for fluorescence imaging.

### 2.6. The Colorimetric/Fluorescent Dual-Mode Detection of Salmonella Based on the NTri-Multi-Apts

The dual-mode colorimetric/fluorescent for the detection of *Salmonella* was developed as follows: 10 μL of the prepared MNP-Apt complexes was added the *Salmonella* solution, followed by incubation at RT for 45 min under gentle shaking. After washing the mixture three times to remove the unbound *Salmonella*, 25 μL of 0.5 μM NTri-Multi-Apt was added and incubated at RT for 45 min to prepare ‘MNPs-bacteria-multiApt’ sandwich structures. After washing the mixture three times again to remove the unbound NTri-Multi-Apt, the precipitate was dissolved in 25 μL of PBS to measure the fluorescence signal via Blue Light LED using the naked eye. The fluorescence intensity of the mixture could be measured using a = multi-mode microplate reader (λ_ex_: 480 nm; λ_em_: 520 nm). Subsequently, an HRP substrate HRP (20 μL TMB) was added, and the reaction was terminated using 20 μL of H_2_SO_4_ (2 M) after 15 min. Finally, the resulting solutions were observed via the naked eye, and the optical density at 450 nm was measured.

### 2.7. The Preparation of Spiked Samples

Milk, eggs, and chicken meat were purchased from Ningbo. To prepare the milk for *Salmonella* spiking, the supernatant layer was isolated following centrifugation to eliminate impurities. For the eggs, the egg whites were obtained and centrifuged, following the sterilization of the eggs by cleaning the shells with 70% (*v*/*v*) ethanol and subsequently breaking them using a sterile fork. Subsequently, the supernatant was diluted 10-fold and set aside for later use. Then, 25 g of chicken meat was added to PBS, followed by homogenization for 2 min. The resulting suspension was then centrifuged at 2000 rpm for 10 min, and the supernatant was collected to serve as the chicken matrix. Finally, the spiked samples were subjected to testing utilizing the proposed detection method.

## 3. Results and Discussion

### 3.1. The Principle of Bacterial Detection

**Scheme 1:**
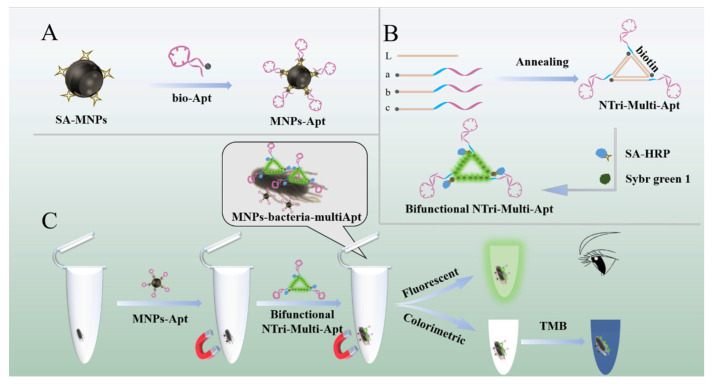
An illustration of the process of preparing (**A**) MNP-Apts and (**B**) bifunctional NTri-Multi-Apts and (**C**) the colorimetric/fluorescence dual-mode detection of *Salmonella* based on the magnetic separation of aptamers and NTri-Multi-Apts.

The mechanism of the DNA-nanotriangle-multivalent-aptamer-assisted colorimetric/fluorescent dual-mode method for the detection of *Salmonella* is shown in Scheme 1. Initially, the aptamer specific for *Salmonella* was immobilized onto the surfaces of magnetic nanoparticals via the biotin–streptavidin interaction to fabricate aptamer magnetic nanoparticles (MNP-Apts) for the isolation of the bacteria (Scheme 1A). Concurrently, three biotin-modified DNA strands (a, b, and c) were meticulously engineered, comprising three distinct segments: the foundational motif (pale brown) for the orchestrated assembly of the DNA nanostructure, an intervening spacer element (blue) for preserving the flexibility of the aptamer, and the aptamer (red) for conferring the requisite binding affinity. Through programmed assembly, these biotin-modified DNA strands were orchestrated onto a lengthy scaffold strand (‘L’, pale brown) to construct the nanotriangle, termed the ‘NTri-Multi-Apt’. This intricate construct facilitated the simultaneous immobilization of multiple horseradish peroxidase (HRP) molecules via biotin–streptavidin interactions and preloaded an extensive quantity of fluorescent-signal-generating indicators in the dsDNAs of the nanotriangle in advance (Scheme 1B). Herein, we adopted Sybr green I as the signal-generating indicator because it can produce a strong fluorescent signal upon intercalation into the minor groove of a triangular dsDNA scaffold and could facilitate the amplification and readout of the fluorescent signal. In the presence of *Salmonella* (Scheme 1C), the target bacteria were separated via the MNP-Apts and subsequently labeled by the NTri-Multi-Apt with high affinity to form an ‘MNPs-bacteria-multiApt’ sandwich complex. The sample exhibited a robust fluorescent emission owing to the presence of the Sybr green I molecules embedded in the dsDNA scaffold. Subsequently, with the addition of a chromogenic substrate, the complex generated a change in color from colorless to a dark blue due to the catalysis of HRP, which can be directly observed using the naked eye. Hence, based on the relationship between the colorimetric/fluorescent signal intensity and the concentration of *Salmonella*, the simple and sensitive detection of *Salmonella* in food samples was achieved.

### 3.2. The Construction of the NTri-Multi-Apt

**Figure 1:**
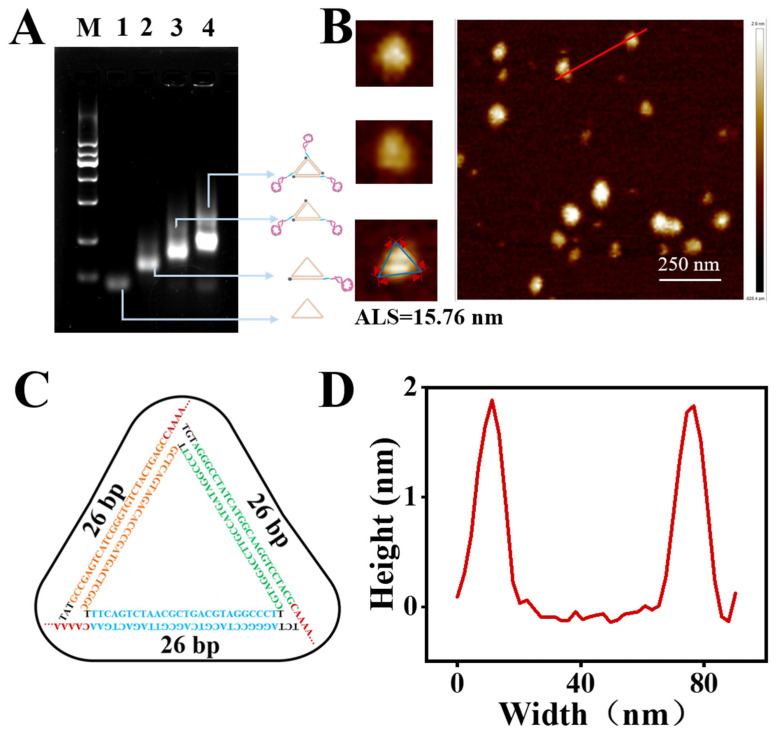
Construction of the NTri-Multi-Apt. (**A**) Electrophoresis images showing the stepwise assembly of the NTri-Multi-Apt (M: Marker, Lane 1: L; Lane 2: L + a; Lane 3: L + a + b; Lane 4: L + a + b + c). (**B**) AFM image of the NTri-Multi-Apt accompanied by the enlarged images. ASL denotes the average side length. (**C**) The structural representation of an NTri-Multi-Apt, including the base sequences of the DNA components. (**D**) The height profile along the red line of Figure 1B.

The assembly of the nanotriangle multivalent aptamer was initially analyzed using electrophoresis for characterization in Figure 1A. The long scaffold strand (L) alone showed the fastest electrophoretic mobility (Lane 1). Upon hybridization with aptamer strands, the mobility gradually decreased as the number of aptamer strands increased, indicating the successful assembly of a monovalent aptamer, divalent aptamer, and trivalent aptamer, respectively. Furthermore, the structural configuration and size of the NTri-Multi-Apt were characterized via atomic force microscopy (AFM). The NTri-Multi-Apt was observed to be of a regular, triangle-like shape with an average side length of ca. 15.76 nm (Figure 1B), which is slightly larger than the theoretical value (8.84 nm, Figure 1C) due to the well-known tip-broadening effect [30,31]. In addition, the cross-sectional profile of the NTri-Multi-Apt in Figure 1D, taken along the red line of Figure 1B, shows that the height of the NTri-Multi-Apt is nearly 2 nm, which is consistent with the theoretical value of dsDNA [32]. Therefore, all the findings substantiated the successful construction of an NTri-Multi-Apt.

### 3.3. The Enhanced Binding Affinity of the NTri-Multi-Apt

**Figure 2:**
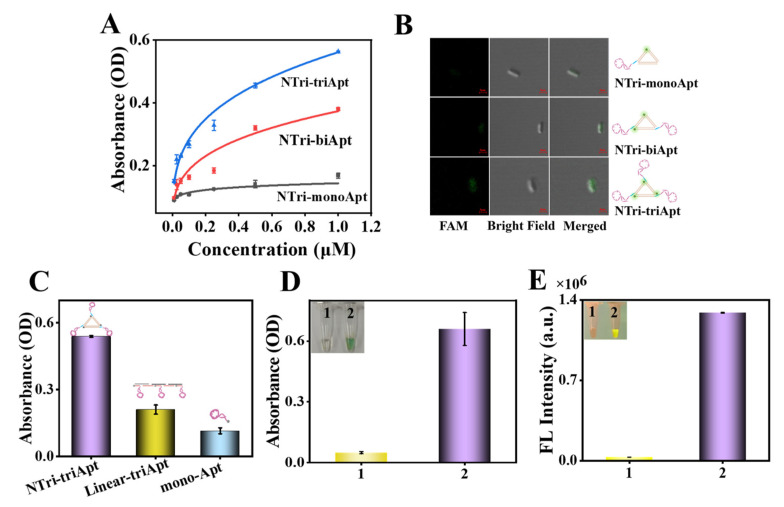
(**A**) The determination of the dissociation constants of the NTri-monoApt, NTri-biApt, and NTri-multiApt. (**B**) A comparison of the binding abilities of the NTri-monoApt, NTri-biApt, and NTri-triApt via confocal microscopy; scale bar: 5 μm. (**C**) A comparison of the binding abilities of the NTri-Multi-Apt, linear trivalent aptamer, and monovalent aptamer. The colorimetric signal (**D**) and fluorescent signal (**E**) without (1) and with (2) *Salmonella*. The insert images are photos of blank and *Salmonella* samples.

The dissociation constants (Kds) of aptamer structures with different valences were determined via a typical ELISA in which the aptamers were used as labeled probes against *Salmonella*. As shown in Figure 2A, the Kds of the NTri-monoApt, NTri-biApt, and NTri-triApt were measured to be 57.32 nM, 43.09 nM, and 11.89 nM, respectively, which were gradually decreased with the improvement of the valences. In particular, the NTri-triApt exhibited a 4.8-fold enhancement in binding affinity over the NTri-monoApt, which means the effect of multivalence was more than the mere summation of the effects of the monovalent aptamer. Furthermore, the FAM-modified NTri-monoApt, NTri-biApt, and NTri-triApt were utilized for visualizing the bacteria via confocal microscopy. As can be evinced from Figure 2B, the *Salmonella* treated with the NTri-triApt (upper) displayed a significantly higher fluorescence intensity compared to the bacteria treated with the same concentrations of NTri-biApt (middle) and NTri-monoApt (lower), which corresponded to the Kds of the aptamer structures. All these results demonstrated the NTri-triApt possessed an enhanced binding affinity. To further confirm the superior affinity of our planar NTri-triApt over the linear one, a linear trivalent aptamer (linear-triApt, Figure S2, Table S3) was fabricated and adopted for the detection of *Salmonella* via an ELISA. As we can see in Figure 2C, due to the enhanced multivalent binding affinity, the colorimetric signals of the samples detected via the trivalent aptamers (planar and linear) were much higher than that of the monovalent aptamer. Notably, the absorbance value of the NTri-triApt showed a 2.5-fold increase over that of the linear-triApt, indicating the higher binding affinity of the NTri-triApt compared to the linear one. We speculated that the aptamers in the plane of the DNA nanotriangle with uniform orientation could fit the surfaces of the bacteria and had high accessibility to their binding sites [33], making them more efficient than the linear-triApt.

Ultimately, the magnetic separation of the aptamers was adopted for capturing *Salmonella* in food samples to reduce the food matrix effect. After the separation, the target bacteria were labeled by the prepared NTri-Multi-Apt for the colorimetric and fluorescent dual-mode detection, and the absorbance value at 450 nm and the fluorescence intensity of Sybr green I were measured. As we can see (Figure 2D), the absorbance values in the presence of *Salmonella* were significantly higher than those without *Salmonella*. In addition, Figure 2E also showed that the *Salmonella* sample presented a distinct fluorescent signal, which is consistent with the result of the colorimetric detection. Therefore, the NTri-Multi-Apt-based colorimetric/fluorescent dual-mode detection of *Salmonella* was successfully established.

### 3.4. The Optimization of the Experimental Conditions

**Figure 3:**
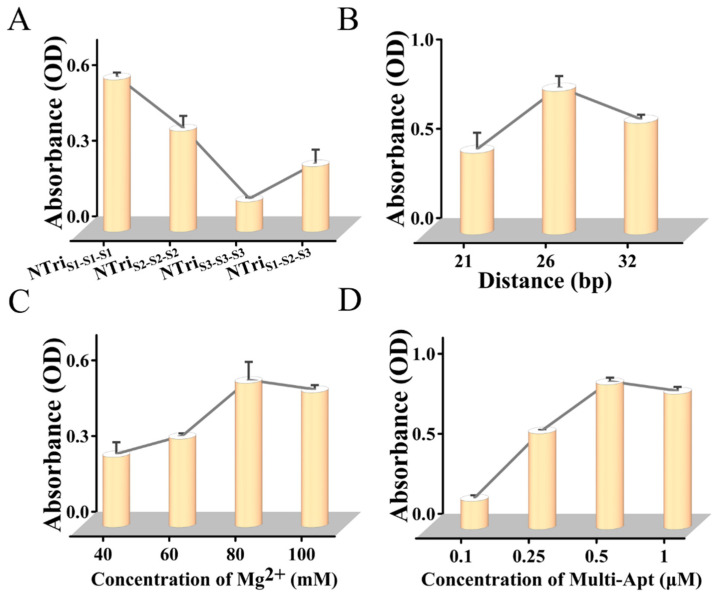
The optimization of the experimental conditions. (**A**) The selection of the aptamer with the best binding affinity among the aptamers S1, S2, and S3 via colorimetric detection. (**B**) Optimizing the spacing between aptamers in the NTri-Multi-Apt. The optimization of the concentration of Mg^2+^ (**C**) and the NTri-Multi-Apt (**D**) in the detection system (*Salmonella*: 10^5^ CFU/mL).

Research has shown that the binding affinity of a multivalent structure is markedly contingent upon the constituent monovalent ligands comprising it [34]. In order to prepare an NTri-Multi-Apt with exceptional binding capability, we judiciously selected three aptamer sequences (Table S4) with high relative binding affinities from previous work [35] for the fabrication of the NTri-Multi-Apt. The respective affinities toward the target bacteria were assessed using the colorimetric method. The results, as depicted in Figure 3A, unequivocally demonstrate a discernible escalation in the absorbance associated with the trivalent aptamer concomitant with an increase in the unit aptamer affinity (S1 > S2 > S3) [36,37]. This observation thereby affirms the explicit positive correlation between the binding avidities of the individual unit aptamers and the multivalent aptamer. In addition, a multivalent structure assembled using heterogeneous aptamers (hetero-Multi-Apt) was reported to exhibit enhanced efficacy compared to its homogeneous aptamer counterparts. This superiority stems from the hetero-Multi-Apt’s capacity to concurrently engage with a diverse array of antigenic epitopes on the surface of a singular target entity, thereby augmenting its overall binding affinity [38,39]. In light of this, we embarked on the construction of a hetero-Multi-Apt by incorporating aptamers S1, S2, and S3 into the DNA nanotriangle framework (designated as NTri_S1-S2-S3_), subsequently employing this construct for the colorimetric detection of *Salmonella*. Regrettably, the absorbance value associated with NTri_S1-S2-S3_ was notably lower in comparison to those attributed to NTri_S1-S1-S1_ and NTri_S2-S2-S2_ and even the average of NTri_S1-S1-S1_, NTri_S2-S2-S2_, and NTri_S3-S3-S3_. This unsatisfactory affinity possibly indicates that the binding sites of these three aptamers were not patterned in the shape or size of our proposed triangular DNA nanostructure. Consequently, the multivalent aptamer assembled using S1 with the strongest affinity was adopted for further study.

The distance between two adjacent aptamers in the multivalent structure is regarded as a critical factor affecting its binding affinity [40]. Consequently, we optimized the distance between adjacent aptamers via precisely controlling the length of the DNA motif (21 bp’s, 26 bp’s, and 32 bp’s, Table S2). Based on the result in Figure 3B, the sample treated with a distance of 26 bp’s exhibited the strongest colorimetric signal, whereas the signals of samples with shorter (21 bp’s) or longer (32 bp’s) distances were considerably weaker. As a result, a distance of 26 bp’s was selected for further investigation. In addition, the ionic strength, particularly the presence of divalent Mg^2+^, could exert an impact on the binding affinity by modulating the secondary structure of the aptamer [41]. Therefore, the concentration of Mg^2+^ was also subjected to optimization (Figure 3C). Notably, we observed a gradual increase in the signal with an elevation of the Mg^2+^ concentration from 40 to 80 mM, reaching its maximum value at 80 mM without any further increase upon the addition of Mg^2+^. Hence, we chose 80 mM as the optimal concentration of Mg^2+^. Last but not least, the NTri-Multi-Apt concentration used for the detection of the target was optimized. As illustrated in Figure 3D, the absorbance value monotonically increased with an increase in the NTri-Multi-Apt concentration from 0.1 to 0.5 µM and then reached a plateau, which indicated that the NTri-Multi-Apt continuously bound on the surface of *Salmonella* and reached saturation at a concentration of 0.5 µM. Thus, a concentration of 0.5 µM was adopted for further study. The effect of pH on the detection was also meticulously assessed in Figure S4. The colorimetric and fluorescent signal exhibited its remarkable highest intensity at a pH of 7.5. Therefore, a pH of 7.5 was used for subsequent experimental research.

### 3.5. The Sensitivity of the Dual-Mode Detection System

**Figure 4:**
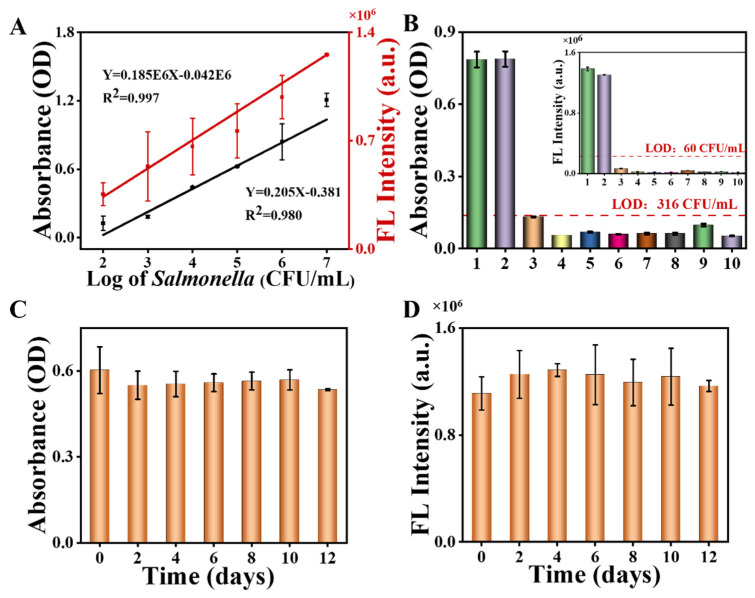
(**A**) An illustration of the linear dynamic range for the quantitative detection of *Salmonella* (black line: colorimetric; red line: fluorescent). (**B**) The specificity of the dual-mode system (1: *Salmonella* typhimurium; 2: MIX (1:1:1:1:1:1:1:1:1); 3: *Salmonella* enteritidis; 4: *S. aureus*; 5: *E. coli O157:H7*; 6: *Vibrio parahaemolyticus*; 7: *Listeria monocytogenes*; 8: *Vibrio alginolyticus*; 9: *Vibrio vulnificus*; 10: negative control). The stability of the colorimetric detection (**C**) and fluorescent detection (**D**) of the dual-mode system.

To evaluate the analytical performance of the quantitative detection of *Salmonella*, we measured the absorbance and fluorescent intensity with different concentrations of *Salmonella* under optimized experimental conditions. In Figure 4A, for the colorimetric assay, we observed that the color changed from colorless to dark blue in a *Salmonella*-concentration-dependent way. The absorbance value was proportional to the logarithm of the *Salmonella* concentration among 1.0 × 10^2^–1.0 × 10^7^ CFU/mL, and the linear regression plot was fitted as *Y* = 0.205 *X* − 0.381 (*X*: the *Salmonella* in log CFU/mL, *Y*: the absorbance value). The limit of detection was calculated to be 316 CFU/mL using the formula 3σ/slope (σ corresponded to the standard deviation of three blank measurements). Likewise, we found a similar enhancement in the fluorescent output via colorimetric detection. The enhanced fluorescence intensity was proportional to the logarithm of the *Salmonella* concentration among 1.0 × 10^2^–1.0 × 10^7^ CFU/mL, and the linear equation of *Y* = 0.185 × 10^6^ *X* − 0.042 × 10^6^ (*X*: the concentration of *Salmonella* in log CFU/mL, *Y*: the signal intensity of fluorescence). Additionally, the fluorescence assay displayed a LOD of 60 CFU/mL, which was superior to several other detections (Table S6). In addition, the fluorescent detection showed a higher sensitivity than the colorimetric method, which aligns with previous findings. [42]. Therefore, it could be stated that this proposed NTri-Multi-Apt-based colorimetric/fluorescent dual-mode method had a relatively high sensitivity for the detection of *Salmonella*.

### 3.6. The Specificity and Stability of the Colorimetric/Fluorescent Dual-Mode System

To verify the specificity of the proposed detection method, some nontarget pathogens including *Salmonella* enteritidis, *E. coli O157:H7*, *S. aureus*, *Listeria monocytogenes*, *Vibrio parahaemolyticus*, *Vibrio alginolyticus*, and *Vibrio vulnificus* were determined using the proposed method. Figure 4B shows that the presence of the target *Salmonella* can lead to very high absorbance and fluorescent signals. Conversely, the signals obtained from the non-target bacterial samples were significantly lower, similar to those of the blank sample. In particular, we also applied the dual-mode system for the detection of a bacterial mixture comprising *Salmonella* typhimurium, *Salmonella* enteritidis, *S. aureus*, *E. coli O157:H7*, *Vibrio parahaemolyticus*, *Listeria monocytogenes*, *Vibrio alginolyticus*, and *Vibrio vulnificus* in a 1:1:1:1:1:1:1:1:1 ratio. The absorbance value and fluorescence signal obtained from this mixed bacterial sample were equivalent to those generated from *Salmonella* typhimurium alone. Furthermore, the signals of *Salmonella* enteritidis, which belongs to the same genus as our target *Salmonella* typhimurium, was slightly higher than the other non-target bacteria but still lower than the LOD (red dashed line) regardless of whether colorimetric or fluorescent detection was used. Therefore, these results illustrate that our proposed method exhibits excellent specificity for *Salmonella* typhimurium.

The stability of the dual-mode system is crucial for its successful utilization and preservation. Therefore, the stability of the NTri-Multi-Apt complexes under varying storage durations (0, 2, 4, 6, 8, and 12 days) was evaluated. As evinced by Figure 4C,D, both the colorimetric and fluorescent signals remained consistent throughout the storage duration with no significant differences, revealing the acceptable stability of the dual-mode assay for *Salmonella* detection.

### 3.7. An Analysis of Salmonella Detection in Food Samples

**Figure 5:**
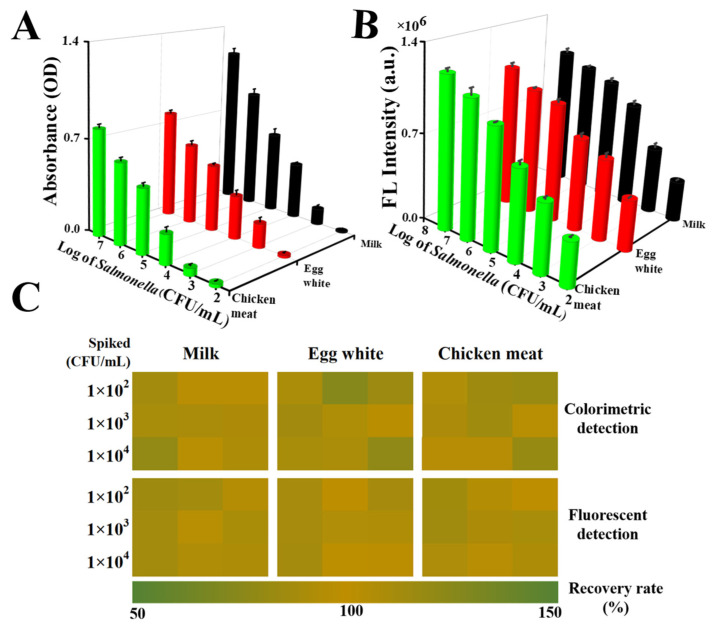
(**A**) The colorimetric signals for *Salmonella* detection in milk (black), egg white (red), and chicken meat (green) with different concentrations. (**B**) Fluorescence signals for *Salmonella* detection in milk (black), egg white (red), and chicken meat (green) with different concentrations. (**C**) A heat map of the recovery rates of the food samples based on the proposed dual-mode method.

The practicability of the proposed colorimetric and fluorescent dual-mode detection method was evaluated using *Salmonella*-spiked food samples. As shown in Figure 5A,B, both the colorimetric signal and fluorescent intensity increased with an increase in the *Salmonella* concentration (10^2^ to 10^7^ CFU/mL) in milk, egg whites, and chicken meat. In addition, the accuracy of the dual-mode detection method was also estimated by measuring the recoveries and coefficients of variation (CVs) of the above-mentioned *Salmonella*-spiked samples (Figure 5C and Table S5). The average recoveries of the colorimetric signal ranged from 92.16% to 116.44%, with a CV of less than 9.71%, and the average recoveries of the fluorescence signal ranged from 93.90% to 112.83%, with a CV of less than 9.53%, for different concentrations of *Salmonella* (1 × 10^2^–1 × 10^4^ CFU/mL), showing satisfactory accuracy for the analysis of *Salmonella*. All these results demonstrate that the multivalent-aptamer-based colorimetric/fluorescent dual-mode method developed for the detection of *Salmonella* in food samples has satisfactory application potential and favorable reproducibility.

## 4. Conclusions

In summary, a colorimetric/fluorescent dual-mode for the determination of the pathogen *Salmonella* was developed via combining aptamer magnetic separation with a bifunctionalized NTri-Multi-Apt. The fabricated NTri-Multi-Apt not only possessed a 1.8-fold enhanced affinity over a monovalent aptamer but was also loaded with numerous colorimetric/fluorescent tags for signal amplification. Due to the dual-functionality of the NTri-Multi-Apt, the proposed method allowed for the simple, stable, and self-calibrated detection of *Salmonella* with satisfactory sensitivity. Furthermore, the proposed method can detect *Salmonella* in food samples with high specificity, accuracy, and stability. Finally, by virtue of the versatility of the aptamer, the application range of our NTri-Multi-Apt-based colorimetric/fluorescent dual-mode method can be extended to various targets other than bacteria. Therefore, this method holds great potential for further applications in the detection of other bacteria, food safety monitoring, or clinical diagnostics.

## Data Availability

No new data were created or analyzed in this study. Data sharing is not applicable to this article.

## Supplementary Materials

The following supporting information can be downloaded at: https://www.mdpi.com/article/10.3390/foods12203853/s1, Refs [43,44,45,46,47,48,49].
