# Supplementary material for: A Colorimetric/Fluorescent Dual-Mode Aptasensor for Salmonella Based on the Magnetic Separation of Aptamers and a DNA-Nanotriangle Programmed Multivalent Aptamer
Source: Foods. 2023 Oct 20;12(20):3853. doi: 10.3390/foods12203853 (PMC10606715; doi:10.3390/foods12203853)
Supplement: Supplementary file 1 [file foods-12-03853-s001.zip › foods-2632854-supplementary.pdf]

Supplementary Materials

A colorimetric/fluorescent dual-mode aptasensor of *Salmonella* based on aptamer magnetic separation and DNA nanotriangle programmed multivalent aptamer

Na Ma[1,2], Mengni Sun[1,2], Hanxing Shi[1,2], Liangliang Xue[1,2], Min Zhang[1,2], Wenge Yang[1,2], Yali Dang[1,2], Zhaohui Qiao[1,2*]

1. College of Food and Pharmaceutical Sciences, Ningbo University, Ningbo 315800, China

2. Zhejiang-Malaysia Joint Research Laboratory for Agricultural Product Processing and Nutrition, Ningbo University, Ningbo 315800, China

[*]Corresponding author

College of Food and Pharmaceutical Sciences, Ningbo University, Ningbo 315800

Email: qiaozhaohui@nbu.edu.cn

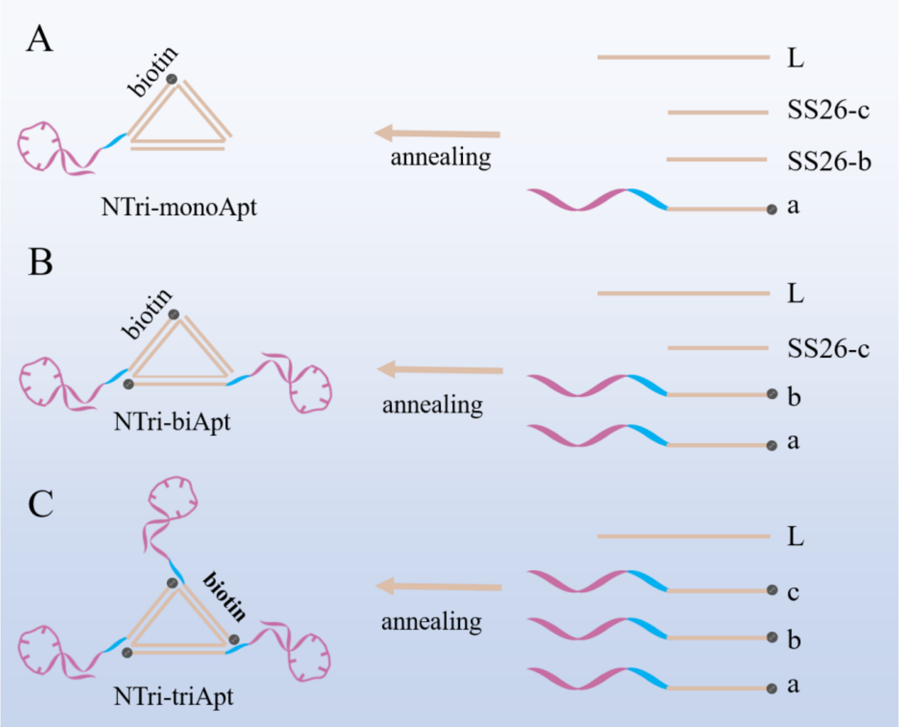

Figure. S1 Illustration of the preparation processes of (A) NTri-monoApt, (B) NTri-biApt, (C) NTri-triApt.

Preparation of linear trivalent aptamers.

All DNA sequences used for linear trivalent aptamers were listed in Table S3. Briefly, eight short ssDNAs were prepared and classified into two categories: linker DNA (1-3 black lines) and spacer DNA (a-b red lines). After hybridization, the spacer DNAs can be sequentially connected through the linker DNA, leaving a gap of 26 bp between each spacer DNA, which just connects with the 26 bp extension fragment of the aptamer. In this way, linear trivalent aptamers were prepared, keeping the aptamer spacing consistent with planar NTri-Multi-Apt.

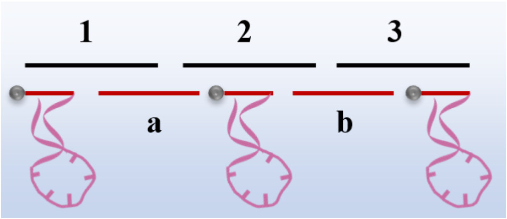

Figure. S2. Schematic design of linear trivalent aptamer.

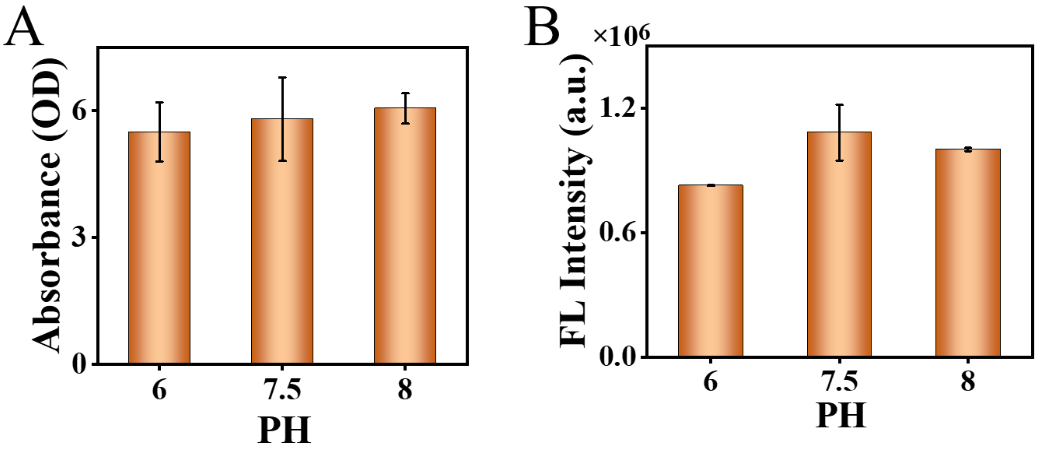

Figure. S3. The effect of pH on the colorimetric (A) and fluorescent (B) detection.

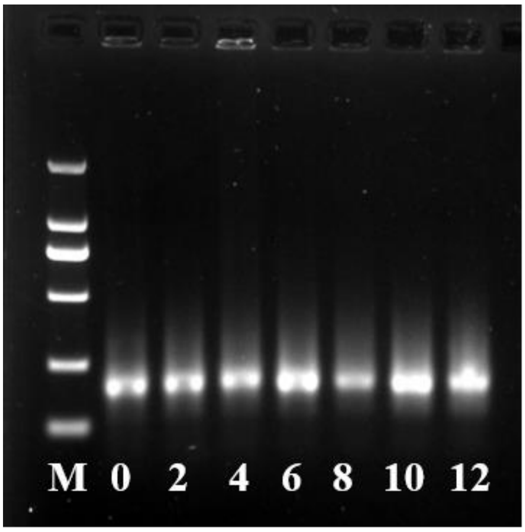

Figure. S4. Agarose gel electrophoresis analysis of NTri-Multi-Apt under different storage durations (0, 2, 4, 6, 8, and 12 days) at 4°C.

Table S1. Sequences information for the nucleic acids of NTri-Multi-Apts in this study.

Name	Sequences (5' to 3')
L	GATGACTCGGCTTTCAGTCTAACGCTGACGTAGGCCCTTCGTAGGACCT TGCCATGATAGGCCCTTGCTCAGTAGACACCC
a	Biotin-TCTAGGGCCTACGTCAGCGTTAGACTGAACAAAACTTGGGCGGTT GGTGTGATGGGCTTTTTTCGTTGGGCCGG
b	Biotin-TATGCCGAGTCATCGGGTGTCTACTGAGCCAAAACTTGGGCGGTT GGTGTGATGGGCTTTTTTCGTTGGGCCGG
c	Biotin-TGTAGGGCCTATCATGGCAAGGTCCTACGCAAAACTTGGGCGGTT GGTGTGATGGGCTTTTTTCGTTGGGCCGG
S26-a	FAM-TCTAGGGCCTACGTCAGCGTTAGACTGAACAAAACTTGGGCGGTT GGTGTGATGGGCTTTTTTCGTTGGGCCGG
S26-b	FAM-TATGCCGAGTCATCGGGTGTCTACTGAGCCAAAACTTGGGCGGTT GGTGTGATGGGCTTTTTTCGTTGGGCCGG
S26-c	FAM-TGTAGGGCCTATCATGGCAAGGTCCTACGCAAAACTTGGGCGGTT GGTGTGATGGGCTTTTTTCGTTGGGCCGG
SS26-b	GCCGAGTCATCGGGTGTCTACTGAGC
SS26-c	AGGGCCTATCATGGCAAGGTCCTACG
Biotin-Apt	biotin-TTTTTTTTTTTTTTTCTTGGGCGGTTGGTGTGATGGGCTTTTTTCG TTGGGCCGG

Table S2. Sequence information for the nucleic acids of NTri-Multi-Apt with different interval lengths in this study.

Name	Sequences (5' to 3')
L21	GATGACTCGTTTCAGTCTAACGCTGACGTAGTCGTAGGACCTTGCCATGATAGTGCTCAGTAGACA
S21-a	Biotin-TCTCTACGTCAGCGTTAGACTGAACAAAACTTGGGCGGTTGGTGTGATGGGCTTTTTTCGTTGGGCCGG
S21-b	Biotin-TATCGAGTCATCTGTCTACTGAGCCAAAACTTGGGCGGTTGGTGTGATGGGCTTTTTTCGTTGGGCCGG
S21-c	Biotin-TGTCTATCATGGCAAGGTCCTACGCAAAACTTGGGCGGTTGGTGTGATGGGCTTTTTTCGTTGGGCCGG
L26	GATGACTCGGCTTTCAGTCTAACGCTGACGTAGGCCCTTCGTAGGACCTTGCCATGATAGGCCCTTGCTCAGTAGACACCC
S26-a	Biotin-TCTAGGGCCTACGTCAGCGTTAGACTGAACAAAACTTGGGCGGTTGGTGTGATGGGCTTTTTTCGTTGGGCCGG
S26-b	Biotin-TATGCCGAGTCATCGGGTGTCTACTGAGCCAAAACTTGGGCGGTTGGTGTGATGGGCTTTTTTCGTTGGGCCGG
S26-c	Biotin-TGTAGGGCCTATCATGGCAAGGTCCTACGCAAAACTTGGGCGGTTGGTGTGATGGGCTTTTTTCGTTGGGCCGG
L32	GATGACTCGGCCAATTTCAGTCTAACGCTGACGTAGGCCCTACCAAATCGTAGGACCTTGCCATGATAGGCCCTACCAAATGCTCAGTAGACACCCATA
S32-a	Biotin-TCTTTTGGTAGGGCCTACGTCAGCGTTAGACTGAACAAAACTTGGGCGGTTGGTGTGATGGGCTTTTTTCGTTGGGCCGG
S32-b	Biotin-TATTTGGCCGAGTCATCTATGGGTGTCTACTGAGCCAAAACTTGGGCGGTTGGTGTGATGGGCTTTTTTCGTTGGGCCGG
S32-c	Biotin-TGTTTTGGTAGGGCCTATCATGGCAAGGTCCTACGCAAAACTTGGGCGGTTGGTGTGATGGGCTTTTTTCGTTGGGCCGG

Table S3. Sequence information for the nucleic acids of linear trivalent aptamer in this study.

Name	Sequences (5' to 3')
1	GCGTCCAGCTCAGGCTACGTACACTTC
2	ATGATGAGGGAGCAGTAGGCGTCGGT
3	CAGGTACGGACAGCGTC
Apt1	Biotin-GAAGTGTACGTAGCCTGATTCTTGGGCGGTTGGTGTGATG GGCTTTTTTCGTTGGGCCGG
Apt2	Biotin-TACTGCTCTTCTTGGGCGGTTGGTGTGATGGGCTTTTTTCG TTGGGCCGG
Apt3	Biotin-CGTACCTGTTCTTGGGCGGTTGGTGTGATGGGCTTTTTTCG TTGGGCCGG
A	GCTGGACGCACCGACGCC
B	CCTCATCATGACGCTGTC

Table S4. Sequence information for the nucleic acids of S1, S2, S3 and the corresponding DNA sequences that make up the NTri-Multi-Apt.

Name	Sequences (5' to 3')
S1	CTTGGGCGGTTGGTGTGATGGGCTTTTTCGTTGGGCCGG
S2	TATGGCGGCGTCACCCGACGGGGACTTGACATTATGACAG
S3	GAGTTAATCAATACAAGGCGGGAACATCCTTGGCGGTGC
L-d	GATGACTCGGCTTTCAGTCTAACGCTGACGTAGGCCCTTCGTAGGA CCTTGCCATGATAGGCCCTTGCTCAGTAGACACCC
S26-1-a	Biotin-TCTAGGGCCTACGTCAGCGTTAGACTGAACAAAACTTGGGCG GTTGGTGTGATGGGCTTTTTCGTTGGGCCGG
S26-1-b	Biotin-TATGCCGAGTCATCGGGTGTCTACTGAGCCAAAACTTGGGCG GTTGGTGTGATGGGCTTTTTCGTTGGGCCGG
S26-1-c	Biotin-TGTAGGGCCTATCATGGCAAGGTCCTACGCAAAACTTGGGCG GTTGGTGTGATGGGCTTTTTCGTTGGGCCGG
S26-2-a	Biotin-TCTAGGGCCTACGTCAGCGTTAGACTGAACAAAATATGGCGG CGTCACCCGACGGGGACTTGACATTATGACAG
S26-2-b	Biotin-TATGCCGAGTCATCGGGTGTCTACTGAGCCAAAATATGGCGG CGTCACCCGACGGGGACTTGACATTATGACAG
S26-2-c	Biotin-TGTAGGGCCTATCATGGCAAGGTCCTACGCAAAATATGGCGG CGTCACCCGACGGGGACTTGACATTATGACAG
S26-3-a	Biotin-TCTAGGGCCTACGTCAGCGTTAGACTGAACAAAAGAGTTAAT CAATACAAGGCGGGAACATCCTTGGCGGTGC
S26-3-b	Biotin-TATGCCGAGTCATCGGGTGTCTACTGAGCCAAAAGAGTTAAT CAATACAAGGCGGGAACATCCTTGGCGGTGC
S26-3-c	Biotin-TGTAGGGCCTATCATGGCAAGGTCCTACGCAAAAGAGTTAAT CAATACAAGGCGGGAACATCCTTGGCGGTGC

Table S5. Recovery of the proposed colorimetric and fluorescent method in *Salmonella*-spiked samples.

Sample	*Salmonella* Spiked (CFU/mL)	Colorimetric			Fluorescent		
		Detected (CFU/mL)	Recovery (%)	CV (%)	Detected (CFU/mL)	Recovery (%)	CV (%)
Milk	1×10^2	98	98.10	6.72	113	112.83	8.54
	1×10^3	1164	116.44	5.74	999	99.89	9.53
	1×10^4	11549	115.49	4.25	10566	105.66	5.42
Egg white	1×10^2	92	92.16	9.71	103	103.25	3.78
	1×10^3	1072	107.18	8.51	1068	106.8	7.8
	1×10^4	10322	103.21	9.36	11085	110.85	9.49
Chicken meat	1×10^2	108	108.63	8.66	94	93.9	7.85
	1×10^3	1097	109.70	4.84	1015	101.45	5.56
	1×10^4	9584	95.84	9.03	10387	103.87	6.57

Table S6. Comparison with different methods of recently developed for *Salmonella* detection.

**Detection method**	**Linear range (CFU/mL)**	**LOD (CFU/mL)**	**Reference**
fluorescent	7×10^3 - 3×10^8	1×10^3	[1]
fluorescent	1503 - 96938	733 and 464	[2]
fluorescent	4.9×10^3 - 4.9×10^7	4.9×10^3	[3]
fluorescent	10^3 - 10^7	10^3	[4]
fluorescent	4.6×10^2 - 4.6×10^7	82	[5]
Colorimetric	1×10^3 - 1×10^8	10^3	[6]
Colorimetric	10^5 - 10^8	10^5	[7]
Colorimetric	8×10^1 - 8×10^4	62	[8]
Colorimetric	$10 – 10^7$	100	[9]
Colorimetric/fluorescent	$1.88 \times 10^4 – 1.88 \times 10^7$	1.88×10^4/3.75×10^3	[10]
Colorimetric/fluorescent	10^2 - 10^7	316/60	This work

References

1. Zhuang, Q.-Q.; He, S.-B.; Jiang, Y.-C.; Huang, K.-Y.; Xu, Y.-Y.; Peng, H.-P.; Deng, H.-H.; Chen, W. Immunofluorescent-aggregation assay based on anti-*Salmonella typhimurium* IgG-AuNCs, for rapid detection of *Salmonella typhimurium*. *Mikrochim Acta* **2022**, *189*, 160, doi:10.1007/s00604-022-05263-z.
2. Srinivasan, S.; Ranganathan, V.; DeRosa, M.C.; Murari, B.M. Label-free aptasensors based on fluorescent screening assays for the detection of *Salmonella typhimurium*. *Anal. Biochem.* **2018**, *559*, 17-23, doi:10.1016/j.ab.2018.08.002.
3. Ding, S.; Hu, H.; Yue, X.; Feng, K.; Gao, X.; Dong, Q.; Yang, M.; Tamer, U.; Huang, G.; Zhang, J. A fluorescent biosensor based on quantum dot–labeled streptavidin and poly-l-lysine for the rapid detection of *Salmonella* in milk. *J. Dairy Sci.* **2022**, *105*, 2895-2907, doi:10.3168/jds.2021-21229.
4. Yang, L.; Li, Y. Quantum dots as fluorescent labels for quantitative detection of *Salmonella Typhimurium* in chicken carcass wash water. *J. Food Prot.* **2005**, *68*, 1241-1245, doi:10.4315/0362-028x-68.6.1241.
5. Yang, X.; Wang, L.; Pang, L.; Fu, S.; Qin, X.; Chen, Q.; Man, C.; Jiang, Y. A novel fluorescent platform of DNA-stabilized silver nanoclusters based on exonuclease III amplification-assisted detection of *Salmonella Typhimurium*. *Anal. Chim. Acta* **2021**, *1181*, 338903, doi:10.1016/j.aca.2021.338903.
6. Wu, W.; Li, J.; Pan, D.; Li, J.; Song, S.; Rong, M.; Li, Z.; Gao, J.; Lu, J. Gold

nanoparticle-based enzyme-linked antibody-aptamer sandwich assay for detection of *Salmonella Typhimurium*. *ACS Appl. Mater. Interfaces* **2014**, *6*, 16974-16981, doi:10.1021/am5045828.

7. Wang, Q.-Y.; Kang, Y.-J. Bioprobes based on aptamer and silica fluorescent nanoparticles for bacteria *Salmonella typhimurium* detection. *Nanoscale Res. Lett.* **2016**, *11*, 150, doi:10.1186/s11671-016-1359-z.
8. Cai, G.; Wang, Y.; Zhang, Y.; Zheng, L.; Lin, J. Magnetorheological elastomer and smartphone enable microfluidic biosensing of foodborne pathogen. *Chin Chem Lett.* **2023**, *34*, 108059, doi:10.1016/j.cclet.2022.108059.
9. Srisa-Art, M.; Boehle, K.E.; Geiss, B.J.; Henry, C.S. Highly sensitive detection of *Salmonella typhimurium* using a colorimetric paper-based analytical device coupled with immunomagnetic separation. *Anal. Chem.* **2017**, *90*, 1035-1043, doi:10.1021/acs.analchem.7b04628.
10. Hu, J.; Jiang, Y.-Z.; Tang, M.; Wu, L.-L.; Xie, H.-y.; Zhang, Z.-L.; Pang, D.-W. Colorimetric-fluorescent-magnetic nanosphere-based multimodal assay platform for *Salmonella* Detection. *Anal. Chem.* **2018**, *91*, 1178-1184, doi:10.1021/acs.analchem.8b05154.
